# Air–liquid interface–centered oxygen engineering in human brain organoids: intact, sliced, and microfluidic extensions

**DOI:** 10.3389/fcell.2026.1809928

**Published:** 2026-05-20

**Authors:** Ubashini Vijakumaran, Anam Anjum, Premala Devaraju, Nurul Husna Abd Razak, Muhammad Fauzi Daud, Izyan Mohd Idris

**Affiliations:** 1 Institute of Medical Science Technology, Universiti Kuala Lumpur (UniKL), Kajang, Selangor, Malaysia; 2 Department of Neurosurgery, University of Maryland School of Medicine, Baltimore, MD, United States; 3 Inborn Errors of Metabolism and Genetics Unit, Nutrition, Metabolism and Cardiovascular Research Centre, Institute for Medical Research (IMR), National Institutes of Health (NIH), Ministry of Health Malaysia, Shah Alam, Selangor, Malaysia

**Keywords:** air–liquid interface (ALI), brain organoids, hypoxia, microfluidics, sliced brain organoid

## Abstract

Brain organoids have become essential *in vitro* models for investigating human brain development, function, and disease, including both unguided cerebral organoids and guided region-specific neural models. However, their utility is fundamentally constrained by oxygen and nutrient diffusion limits inherent to closed three-dimensional architectures. As organoids increase in size and complexity, restricted oxygen delivery induces metabolic stress, disrupts progenitor dynamics, impairs neuronal maturation, and promotes the formation of hypoxic or necrotic cores, thereby limiting developmental fidelity and long-term experimental stability. This review synthesizes current insights into hypoxia and metabolic stress in brain organoid systems, with an emphasis on the physical determinants of gas exchange and their biological consequences. We critically evaluate engineering strategies developed to overcome diffusion-related constraints, focusing on air–liquid interface (ALI) culture and organoid slicing approaches and emerging ALI–microfluidic platforms that integrate controlled perfusion and geometric confinement to actively regulate mass transport. ALI culture improves surface oxygenation while preserving intact tissue architecture, supporting extended viability and functional maturation in intact organoids and assembloids. Sliced organoid platforms directly expose internal tissue compartments, substantially reducing diffusion distances and enabling uniform metabolic conditions that facilitate advanced neuronal differentiation, circuit-level maturation, and high-resolution functional interrogation. Complementing these diffusion-based strategies, ALI–microfluidic systems further enhance metabolic stability and enable dynamic environmental control, scalable organoid production, and integrated electrophysiological assessment under physiologically regulated conditions. By comparing the advantages and limitations of ALI-based systems, this review highlights how oxygen-engineering strategies reshape tissue organization, maturation trajectories, and experimental accessibility, advancing the physiological relevance of human brain organoid models.

## 1 Introduction

Brain organoid, including both unguided organoids and guided region-specific neural organoid models, are three-dimensional (3D) neural tissue constructs derived from human pluripotent stem cells (hPSCs) that recapitulate essential aspects of early brain development, including neurogenesis, regional specification, and cell-type diversification (Lancaster and Knoblich, 2014; Chen et al., 2022). Compared to conventional two-dimensional monolayer cultures, brain organoids self-organize into layered structures resembling the fetal cerebral cortex and generate diverse neural populations, such as progenitors, neurons, astrocytes, and oligodendrocyte precursors (Corrò et al., 2020). This complexity has made organoids a transformative platform for modelling human neurodevelopmental disorders (Shaikh et al., 2025) and for preclinical drug testing, particularly in neurodegenerative and mitochondrial diseases (Liang, 2024; Ramani et al., 2024), offering new hope for therapeutic discovery in conditions that have long lacked effective treatments. However, despite their promise, conventional brain organoid cultures face major challenges. Absence of intrinsic vasculature one of the major challenges which forces oxygen and nutrient delivery to depend entirely on passive diffusion. This limitation ultimately restricts their growth and maturation (Aili et al., 2024). As organoids grow in size, diffusion becomes inefficient beyond a few hundred micrometres, causing hypoxia, core necrosis, and central cell death that halt further maturation (Pagliaro et al., 2025). This limits the development of the blood-brain barrier (BBB) and its neural-vascular homeostasis (Kistemaker et al., 2025).

**FIGURE 1:**
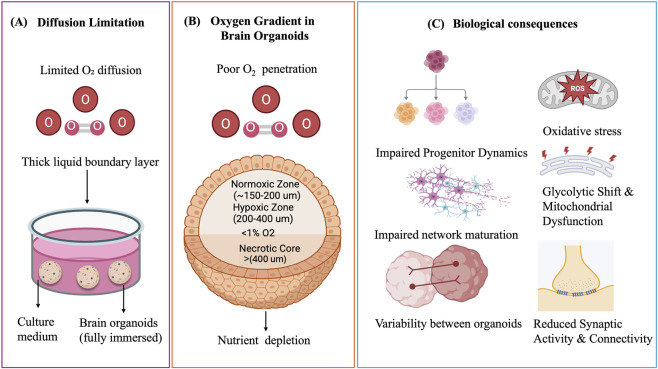
Oxygen diffusion limitation in brain organoids. **(A)** Submerged culture creates a thick liquid boundary layer that restricts O_2_ diffusion. **(B)** This results in spatial oxygen gradients, with normoxic outer layers, hypoxic intermediate regions, and a necrotic core. **(C)** Consequences include oxidative stress, metabolic dysfunction, impaired progenitor dynamics, reduced network maturation, and decreased synaptic connectivity.

Moreover, this causes batch-to-batch variability, heterogeneity in differentiation, and incomplete representation of non-neuronal cell types (e.g., pericytes, microglia, endothelial cells), further constraining reproducibility and fidelity (Urrestizala-Arenaza et al., 2024). Paşca et. 2019 have reported oxygen deprivation impairs cortical development by damaging intermediate progenitor cells through unfolded protein response activation, a defect that can be prevented with a small-molecule modulator (Paşca et al., 2019). Brain organoids exhibit elevated hypoxia-inducible factor-1 (HIF-1) expression due to oxygen limitation in their cores (Cakir et al., 2019) and have been reported has express stress markers that reflected electron transport dysfunction, metabolic and endoplasmic reticulum stress (Bhaduri et al., 2020; Andrews and Kriegstein, 2022). Importantly, while most brain organoids are cultured under dynamic conditions using orbital shakers or spinning bioreactors that enhance convective mixing and improve oxygen availability at the organoid surface, these systems do not overcome the intrinsic limitation of oxygen diffusion within dense three-dimensional tissue. As organoids increase in size and cellular complexity, oxygen transport remains diffusion-limited, leading to the formation of hypoxic gradients and necrotic cores despite continuous agitation. Thus, although dynamic culture systems delay the onset of hypoxia, they are ultimately insufficient to sustain long-term viability and maturation, highlighting the need for alternative strategies that directly enhance oxygen penetration into the tissue. To provide a conceptual framework for these limitations, Figure 1 illustrates the progression from diffusion-restricted oxygen transport to the establishment of spatial oxygen gradients within brain organoids and their downstream biological consequences. Conventional dynamic culture systems, including orbital shakers, spinning bioreactors, and emerging 3D-printed bioreactor platforms, primarily enhance oxygen availability through convective mixing and improved surface gas exchange. However, these systems do not eliminate diffusion limitations within dense three-dimensional tissues, resulting in persistent hypoxic gradients and incomplete waste removal in the organoid core. In addition, shear stress, flow heterogeneity, and system-specific design variations can influence culture stability and reproducibility across platforms.

Recent innovations such as the air–liquid interface (ALI) systems provide promising solutions to this problem. By physically exposing one surface of the organoid to air and reducing diffusion distances through tissue sectioning, these approaches significantly enhance oxygen penetration, nutrient exchange, and waste removal. Consequently, they support long-term viability, improved neuronal network activity, and extended tissue maturation (Giandomenico et al., 2019; Qian et al., 2020). Thus, we aimed to explore how air–liquid interface (ALI)–based culture strategies, including sliced brain organoid systems and emerging microfluidic-assisted platforms, overcome the intrinsic oxygenation limitations of conventional three-dimensional models. By enhancing gas and nutrient exchange through surface exposure, reduced diffusion distance, and controlled perfusion, these approaches establish more physiologically relevant microenvironments that support prolonged tissue survival, neuronal maturation, and functional network development. This review highlights the engineering principles, culture methodologies, and biological outcomes of ALI-centred oxygenation strategies, emphasising intact and sliced organoid platforms, and discusses microfluidic extensions that further improve oxygen delivery, metabolic stability, and experimental accessibility.

## 2 Oxygen gradients in native brain versus organoids

Oxygen serves as a critical substrate for cellular energy production and metabolism, fundamentally governing growth, development, and survival across most life forms. Despite an atmospheric oxygen tension of 156 mmHg, physiological tissue oxygen levels are substantially lower, a phenomenon particularly evident in the brain, which accounts for approximately 20% of total body oxygen consumption, despite representing only 2% of body mass (Masamoto and Tanishita, 2009; Panchision, 2009; Liu et al., 2025). This demand is met by an incredibly dense and hierarchical neurovascular unit (NVU), ensuring that no individual cell is more than a few microns away from a constant supply of oxygenated blood. In contrast, brain organoids are avascular three-dimensional structures that rely entirely on passive diffusion for oxygen and nutrient delivery from the surrounding culture medium, creating severe oxygen gradients that fundamentally constrain their development and size (Giandomenico et al., 2021; Acharya et al., 2024). Brain organoids can expand to 3–4 mm diameter, but only cells within approximately 200–400 µm from the surface receive sufficient oxygen and nutrients via diffusion (Pham et al., 2018) This diffusion limitation results in more than 50% of cells located 150–600 μm from the surface becoming hypoxic or necrotic by day 60 of culture (Adlakha, 2023). Computational modelling of oxygen diffusion and consumption reveals fundamental biophysical constraints on organoid scalability. Mathematical frameworks coupling Michaelis-Menten kinetics with diffusion dynamics predict that homogeneous neuronal spheres would reach viability limits at approximately 1.4 mm diameter due to core hypoxia (McMurtrey, 2016). However, brain organoids frequently exceed 2–4 mm through architectural self-organisation: metabolically active neurons localise to outer cortical layers with high surface area for gas exchange, while inner regions harbour less active progenitors or fluid-filled cavities (McMurtrey, 2016; Acharya et al., 2024). Despite this spatial optimisation, sustained growth beyond 4–5 mm remains challenging, as steep oxygen gradients ultimately create near-anoxic cores and relatively normoxic superficial zones (McMurtrey, 2016; Sedlack et al., 2022). A recent multi-omic analysis by Liu et al. (2025) has identified a “critical neurogenic window” between weeks 4 and 6 of culture. During this period, organoids exhibit a timed elevation in intra-organoid oxygen tension that coincides with rapid neurogenesis and the expansion of the cortical plate (CP) (Liu et al., 2025). This suggests that early neural development is not just a passenger to oxygen availability but is actively shaped by it. When this timed elevation is suppressed either through environmental hypoxia or genetic silencing of oxygen-carrier genes like neuroglobin (NGB), the organoid fails to achieve the necessary metabolic homeostasis for normal development (Liu et al., 2025).

### 2.1 Hypoxia-induced alterations

When the metabolic demand of the growing brain organoids exceeds the rate of oxygen diffusion, a state typically reached by day 35 of culture, the tissue undergoes profound and often irreversible pathological shifts. The most immediate molecular indicator of this stress is the stabilisation and nuclear localisation of Hypoxia-Inducible Factor 1-alpha (HIF-1α). Kim et al. (2021) demonstrated that under 1% oxygen conditions, HIF-1α triggers a pro-apoptotic signalling cascade, resulting in a significant increase in cleaved Caspase-3 (c-Cas3) and c-PARP expression, primarily within the organoid’s deep layers (Kim et al., 2021). The consequences of chronic hypoxia extend beyond simple cell death; they fundamentally reshape the organoid’s cytoarchitecture. Gaston-Breton et al. (2023) note that hypoxic injury in brain organoids is characterised by a thinning of the neuronal layer, disruption of apical surface adherent junctions, and an abnormal enlargement of the ventricular lumen. Furthermore, hypoxia preferentially affects specific cell populations. For an instant, research indicates that intermediate progenitors (TBR2+) and outer radial glia (oRG) are particularly vulnerable to low oxygen levels (Gaston-Breton et al., 2023; Liu et al., 2025). This vulnerability leads to a reduction in the “transit-amplifying” cell pool, effectively stalling the expansion of the human-specific cortical architecture. From a genomic perspective, Umans and Gilad (2025) have shown that oxygen-induced stress reveals context-specific gene regulatory effects that are otherwise “hidden” under normoxic conditions (Umans and Gilad, 2025). Their single-cell RNA sequencing data revealed that hypoxia alters the transcriptome of nearly all brain cell types, particularly affecting genes involved in DNA damage repair and cellular metabolism. This suggests that the hypoxic core of an organoid is not merely “dead” tissue but is a pathologically altered environment where gene-by-environment (GxE) interactions may potentially skew the results of drug screening or disease modelling experiments.

### 2.2 Energy metabolism and developmental mismatch

The metabolic constraints of brain organoids culture induce a significant “developmental mismatch” between the *in vitro* model and the *in vivo* fetal brain. In native development, the brain undergoes a metabolic transition: early-stage neural stem cells rely heavily on anaerobic glycolysis, but as neurons differentiate and mature, they shift toward more energy-efficient oxidative phosphorylation (McMurtrey, 2016). In brain organoids, the limited oxygen access forces cells to remain in a “glycolytic trap.” Because oxidative phosphorylation is oxygen-dependent, mature neurons in the organoid core cannot meet the high ATP demands required for complex processes such as axonal outgrowth, dendritic arborization, and synaptic plasticity (Acharya et al., 2024). This energy deficit is a primary driver of the relative immaturity observed in most HBO models, which rarely progress beyond the equivalent of the early second trimester of human gestation (Gaston-Breton et al., 2023). Research by Kim et al. (2021) provides a critical nuance to this mismatch through reoxygenation experiments. They found that while returning a hypoxic organoid to normoxia (21% O2) could restore cell proliferation, as evidenced by an increase in Ki67+ proliferating cells, it failed to restore cell maturation (Kim et al., 2021). Post-mitotic projection neurons (TBR1+) remained significantly depleted even after reoxygenation, suggesting that the complex, energy-expensive transition from progenitor to mature neuron is irreversibly disrupted by prior hypoxic stress.

Furthermore, the lack of convection in standard organoid culture prevents the effective removal of metabolic catabolites. As observed in studies of thick brain slices, the accumulation of waste products like lactic acid and CO2 further acidifies the microenvironment, exacerbating cellular stress and promoting premature differentiation (Rambani et al., 2009; Gaston-Breton et al., 2023). This metabolic entrapment explains why brain organoids often exhibit a high density of early-born deep-layer neurons but fail to develop the more complex, late-born superficial cortical layers that define the human neocortex. To address these metabolic bottlenecks, the engineering of oxygen access has become a paramount goal in the field. Methods such as slicing and air-liquid interface (ALI) culture significantly reduce the diffusion distance. Chartrain et al. (2025) demonstrated that routinely cutting organoids to reduce their diameter improves nutrient and gas diffusion, leading to a measurable increase in cell proliferation and a reduction in the necrotic core (Chartrain et al., 2025). By bypassing the physical constraints identified by McMurtrey (2016) (McMurtrey, 2016), these engineered approaches allow brain organoids to maintain oxidative metabolism for longer periods, effectively bridging the gap between embryonic-like cellular aggregates and functional, mature neural tissues.

## 3 Physical limitations of three-dimensional organoid

A fundamental limitation of three-dimensional (3D) organoid cultures is restricted transport of oxygen, nutrients, and metabolic waste, arising from their avascular and closed architecture. Similar to other multicellular aggregates, brain organoids rely almost exclusively on passive diffusion from the surrounding medium to meet metabolic demands, a process that becomes increasingly inefficient as tissue size and cellular density increase (Lin and Chang, 2008; Mehta et al., 2012). Unlike two-dimensional monolayer cultures, where cells are uniformly exposed to nutrients and oxygen, organoids grow as compact spherical or semi-spherical tissues, inherently generating spatial gradients in oxygen tension, nutrient availability, and waste accumulation (Nowacka et al., 2021). These non-physiological gradients create distinct microenvironments within the organoid. Cells located near the surface experience relatively adequate oxygen and nutrient supply and efficient waste removal, whereas cells in deeper regions are exposed to progressively lower oxygen tension and accumulation of metabolic by-products (Radisic et al., 2006). As a result, diffusion limitations impose a size-dependent constraint on organoid growth and maturation, with oxygen and nutrient delivery becoming insufficient beyond a few hundred micrometres from the surface. Classical experimental and theoretical studies using multicellular aggregates have demonstrated that oxygen penetration is largely restricted to outer cell layers, leading to reduced viability and metabolic stress toward the core (Mueller-Klieser et al., 1986). These principles directly translate to brain organoids, which frequently exceed diffusion limits during prolonged culture. As organoid size increases, diffusion-driven gradients give rise to spatially segregated zones, including a viable peripheral rim and hypoxic or necrotic central regions. Indeed, cerebral organoids several hundred micrometres to millimetres in diameter commonly develop necrotic cores surrounded by metabolically active outer layers, a feature consistently observed across brain organoid models (Curcio et al., 2007; Däster et al., 2017). Such diffusion-limited pathology restricts long-term viability, disrupts neurodevelopmental trajectories, and contributes to batch-to-batch heterogeneity in organoid cultures.

### 3.1 Diffusion vs. consumption kinetics (Fick’s law context)

Oxygen transport within brain organoids is governed by passive diffusion driven by concentration gradients, whereby oxygen moves from regions of higher availability at the tissue surface toward regions of lower availability in the interior. Early studies on oxygen transport in biological tissues established that diffusion alone governs oxygen supply in avascular systems (Hill, 1928) a condition that directly applies to cerebral organoids. Under steady-state conditions, oxygen flux (F) can be described by Fick’s first law:

F=D×ΔC/Δx

where D is the diffusion coefficient of oxygen in the culture medium, ΔC is the concentration difference, and Δx is the diffusion distance. Importantly, oxygen availability in organoid cultures is constrained not only by incubator oxygen levels but also by diffusion through the overlying culture medium. Place et al. (2017) demonstrated that increased medium height substantially reduces oxygen flux, resulting in pericellular oxygen tensions far below expected values. Because the effective diffusion coefficient of oxygen in serum-supplemented media is poorly defined and influenced by viscosity and composition, oxygen delivery within 3D organoid systems remains difficult to precisely control or predict (Place et al., 2017). These physical constraints explain why strategies that reduce diffusion distance or eliminate liquid diffusion barriers, such as air–liquid interface (ALI) culture, are particularly effective for improving oxygen delivery in organoids.

### 3.2 Diffusion–reaction dynamics in brain organoids

While Fick’s first law describes steady-state diffusion, oxygen transport in brain organoids is inherently dynamic and strongly influenced by cellular consumption. This behaviour is captured by diffusion–reaction models derived from Fick’s second law, incorporating reaction terms that represent oxygen uptake by metabolically active cells (Greenspan, 1972). Such diffusion reaction frameworks have been widely applied to predict oxygen distributions in organoids, demonstrating how the balance between oxygen supply and cellular demand generates spatially heterogeneous microenvironments. These models consistently predict the emergence of viable outer regions, hypoxic intermediate zones, and necrotic cores as organoid size increases, closely mirroring experimental observations in cerebral organoid cultures (Greenspan, 1972; Grimes et al., 2014). Together, these findings establish oxygen limitation not as a technical artifact, but as a fundamental physical constraint of closed, avascular 3D organoid systems.

### 3.3 Key parameters affecting gas exchange: radius, density, and viscosity

Key parameters governing gas exchange in organoids include tissue radius, cellular packing density, and the physical properties of the surrounding culture medium. Increasing organoid size proportionally extends diffusion distance, exacerbating oxygen gradients and reducing oxygen partial pressure toward the core. Experimental and computational studies in metabolically active spheroid and organoid models, including hepatocyte and neural tissues, demonstrate that aggregates reaching diameters of several hundred micrometres exhibit pronounced central oxygen depletion associated with reduced viability and metabolic function (Glicklis et al., 2004; Curcio et al., 2007). Beyond geometric factors, oxygen availability within brain organoids is shaped by cellular microarchitecture and metabolic demand. Dense cellular packing increases local oxygen consumption, whereas reduced packing density creates intercellular spaces that facilitate oxygen and nutrient transport toward inner regions (Murphy et al., 2017). Diffusion–reaction models incorporating Michaelis–Menten kinetics further indicate that oxygen uptake depends on local cellular activity and microenvironment rather than diffusion distance alone (Wagner et al., 2011). In addition, culture medium properties modulate gas exchange, as higher serum content increases medium density and viscosity, reducing oxygen diffusivity (Poon, 2022). Dynamic culture configurations, including agitation-based systems and bioreactors, partially mitigate these limitations by enhancing convective mass transfer and oxygen renewal at the tissue surface (Franchi-Mendes et al., 2021; Kim et al., 2025).

### 3.4 Air–liquid interface (ALI)–Based oxygenation platforms

#### 3.4.1 Sliced-organoid

In sliced-organoid culture, mature cerebral or region-specific organoids are embedded and sectioned with a vibratome into 200–500 µm-thick live slices, which are then transferred onto porous membrane inserts and maintained at the air–liquid interface. This organotypic-style configuration exposes the previously hypoxic interior of the organoid directly to both oxygen and nutrients supplied from the air-exposed surface and the medium below, thereby alleviating the diffusion limitations that constrain intact 3D spheroids. As a result, metabolic stress is markedly reduced, and progressive necrosis within the organoid core is prevented, leading to improved survival of neuronal progenitors, differentiating neurons, and glial populations, as well as more stable preservation of developmental patterning (Giandomenico et al., 2021; Petersilie et al., 2024). Giandomenico and colleagues first demonstrated that cerebral organoid slices cultured at ALI (ALI-COs) exhibit dramatically enhanced neuronal survival and maturation compared with fully submerged organoids. These cultures develop long-range axonal projections, fibre-tract-like bundles, and functionally active neuronal networks, evidenced by spontaneous synaptic activity and electrophysiological responses, that persist for many months, enabling long-term functional and electrophysiological studies of human brain tissue *in vitro* (Giandomenico et al., 2019; Giandomenico et al., 2021).

Qian et al. further showed that forebrain organoid slices maintained at ALI display advanced neuronal differentiation and electrophysiological signatures resembling late-fetal human brain activity, highlighting the ability of this platform to support prolonged circuit-level maturation (Qian et al., 2020).

More recently, standardized cortical brain organoid slice (cBOS) platforms have refined this approach with optimized slicing, mounting, and media formulations, enabling reproducible long-term culture of metabolically stable, highly viable cortical tissue for up to 8–10 months (Petersilie et al., 2024). These systems support astrocyte maturation, synaptic connectivity, axonal tract formation, and functional responses to metabolic stress, making ALI-sliced organoids particularly powerful for studying late developmental stages, neurodevelopmental disorders, and neurodegenerative processes in a human-relevant tissue context.

### 3.5 Intact organoids and assembloid

ALI platforms are also applied to intact organoids and assembloids, although with a different biological objective. In this configuration, whole 3D tissues are placed on porous membrane inserts so that their basal surface remains in contact with medium while the apical surface is exposed to air, creating a partially submerged environment that improves oxygenation and optical accessibility without physically opening the tissue core (Li et al., 2016). This approach is particularly useful for live imaging and electrophysiological recordings, but it does not eliminate diffusion barriers to the same extent as vibratome slicing. Birey and Paşca employed Transwell-based ALI culture to partially flatten human forebrain assembloids, enabling high-resolution imaging of interneuron migration and functional network dynamics while maintaining tissue integrity (Birey and Paşca, 2022). Consistent with this approach, Stoppini et al. developed a porous Strip-MEA platform compatible with ALI culture, enabling long-term electrophysiological monitoring of intact human iPSC-derived 3D neural tissues. This system supports rapid recovery of spontaneous network activity following ALI transfer and allows functional recording from both surface and internal tissue layers, demonstrating that ALI-based platforms can sustain metabolically viable, electrically active intact organoids without microfluidic perfusion (Stoppini et al., 2024). While intact-ALI platforms improve oxygenation and accessibility, they retain a closed 3D architecture and therefore complement, rather than replace, sliced-organoid ALI systems for studies requiring deep tissue access, long-range axonal organization, and full metabolic rescue.

### 3.6 ALI–microfluidic extensions

Building on conventional air–liquid interface (ALI) configurations, emerging microfluidic-assisted ALI platforms introduce controlled perfusion and geometric confinement to further enhance oxygen and nutrient delivery while enabling dynamic regulation of the tissue microenvironment. In these systems, intact organoids or ALI-mounted neural tissues are integrated into microphysiological devices that combine atmospheric oxygen exposure with continuous or pulsatile media flow, effectively coupling surface oxygenation with convective mass transport (Castiglione et al., 2022). This hybrid configuration reduces boundary-layer limitations, improves waste removal, and allows more precise control over biochemical and oxygen gradients compared to static ALI culture alone. Recent studies highlight the functional advantages of these platforms. Ao et al. developed an ALI-integrated microfluidic system that supports the scalable generation of size-uniform cerebral organoids with reduced hypoxia and improved electrophysiological maturation. Using this platform, they demonstrated that prenatal Δ^9^-tetrahydrocannabinol (THC) exposure impairs neuronal differentiation and neurite outgrowth, underscoring the utility of ALI–microfluidic devices for reproducible and higher-throughput neural tissue modelling (Ao et al., 2020). Similarly, Tian et al. established an ALI-integrated human midbrain organoid platform incorporating a perfusable support layer, which enhanced oxygen and nutrient delivery and enabled direct interfacing with microelectrode arrays for functional assessment. This system supported stable long-term electrophysiological activity and revealed that prenatal perfluorooctane sulfonate (PFOS) exposure disrupts neuronal network maturation and dopamine-related signalling (Tian et al., 2024).

**FIGURE 2:**
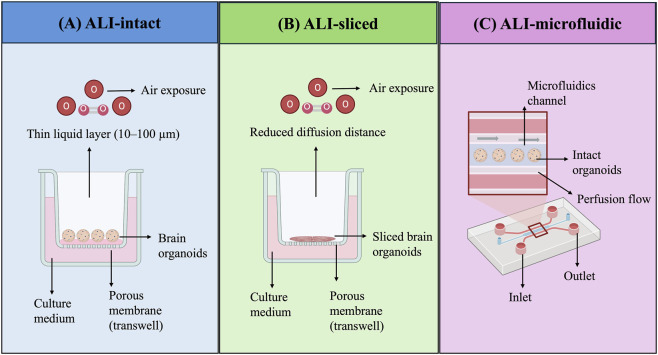
ALI-based strategies for improving oxygen delivery in brain organoids. **(A)** Intact organoids cultured at the air–liquid interface on porous membranes. **(B)** Sliced organoids with reduced diffusion distance for improved oxygen penetration. **(C)** Microfluidic ALI platform integrating intact organoids with perfusion flow to enhance mass transport and microenvironmental control.

Despite these advances, ALI–microfluidic platforms remain technically complex and are not yet standardised across laboratories. While perfusion and geometric confinement partially alleviate diffusion-limited hypoxia, intact three-dimensional constructs still impose intrinsic limits on uniform oxygen distribution, particularly in larger tissues (Castiglione et al., 2022). These ALI-based strategies, including intact, sliced, and microfluidic configurations, are summarised in Figure 2, highlighting their distinct mechanisms for improving oxygen delivery in brain organoids, with their functional characteristics further detailed in Table 1.

**TABLE 1:** Air–liquid interface (ALI)–based strategies for long-term culture and functional analysis of human brain organoids.

Reference	Tissue type	Physical configuration	Culture duration	Compared to submerged	Key findings
Giandomenico et al. (2019)	Cortical cerebral organoids (ALI-COs)	Sliced	Up to ∼6 months	Improved	ALI slice culture improved oxygenation, reduced necrotic cores, enhanced neuronal survival and maturation, promoted long-range axonal growth, and supported functional synaptic activity
Giandomenico et al. (2021)	Cortical cerebral organoids	Sliced	>6 months	Improved	Demonstrated sustained neuronal viability, increased network complexity, reproducible cortical layer-like organization, and long-term electrophysiological stability
Qian et al. (2020)	Forebrain organoids	Sliced	∼3–5 months	Improved	ALI slice culture promoted advanced neuronal differentiation and enabled electrophysiological recordings resembling late-fetal brain activity
Petersilie et al. (2024)	Cortical brain organoids (cBOS)	Sliced	Up to ∼8–10 months	Improved	Long-term ALI supported high neuronal viability without necrotic cores, maturation of synaptically connected neurons and astrocytes, axonal fiber tract formation, and functional assessment via patch-clamp, calcium imaging, and ATP imaging under metabolic stress
Birey and Paşca (2022)	Forebrain assembloids (hCS + hSS)	Intact	∼3–6 months	Improved	ALI-flattened assembloids supported long-term viability, enabled interneuron migration between regions, and allowed functional network analysis via live calcium imaging
Stoppini et al. (2024)	Human iPSC-derived 3D neural tissues	Intact	≥8 weeks (MEA on ∼17–18-month-old tissues)	Not assessed	ALI-compatible MEA platform enabled stable, long-term electrophysiological monitoring of highly mature human 3D neural tissues
Tian et al. (2024)	Human midbrain organoids	Intact and Microfluidics	∼1–5 months	Not assessed	ALI-enhanced microphysiological system enabled MEA-based functional monitoring and revealed PFOS-induced neurodevelopmental and neurotoxic effects
Ao et al. (2020)	Human cerebral organoids	Intact and Microfluidics	1–3 months	Improved	ALI-integrated microfluidic platform supported size-uniform cerebral organoids with reduced hypoxia and revealed THC-induced deficits in neuronal maturation and network activity

## 4 Comparative functional outcomes of ALI-Centred oxygenation strategies

The development of air–liquid interface (ALI)–based culture strategies, including intact ALI, sliced organoid systems, and emerging ALI–microfluidic platforms, represents a major advance in addressing diffusion-related constraints in conventional three-dimensional organoid models. Although these approaches share the goal of improving oxygen availability and tissue viability, they operate through distinct physical and biological mechanisms that differentially influence tissue architecture, maturation trajectories, and experimental accessibility (Ji and Sun, 2024; Chartrain et al., 2025; Luna et al., 2025).

### 4.1 Oxygenation and diffusion dynamics

Oxygen diffusion remains a central constraint in organoid biology where intact spherical organoids often develop necrotic cores as their size exceeds diffusion limits (Urrestizala-Arenaza et al., 2024). ALI cultures address this constraint by exposing the apical or dorsal surface of the organoid or tissue construct directly to atmospheric oxygen, drastically increasing available oxygen tension while maintaining nutrient supply from basal media (Sekiya et al., 2019). This dual exposure creates more physiologically relevant oxygen gradients and sustains viability in larger and more complex tissues (Giandomenico et al., 2019). In contrast, organoid slicing enhances oxygenation through geometric modification. By sectioning organoids into thin slices typically 100–300 μm, oxygen and nutrients can penetrate evenly throughout the entire tissue. This method reduces metabolic stress and prevents the formation of hypoxic zones altogether. The result is a uniformly oxygenated tissue microenvironment, allowing synchronised cellular responses and improved metabolic efficiency across all layers (Qian et al., 2020). Complementing these passive diffusion based strategies, emerging ALI–microfluidic platforms further enhance oxygenation by integrating controlled perfusion and geometric confinement, coupling atmospheric oxygen exposure with convective mass transport to actively regulate nutrient delivery and waste removal. These hybrid systems partially alleviate diffusion-limited hypoxia in intact constructs while enabling dynamic control of the tissue microenvironment, supporting functional maturation under physiologically relevant conditions (Ao et al., 2020; Tian et al., 2024).

### 4.2 Structural integrity and architectural considerations

ALI systems largely preserve the native 3D structure of organoids, making them particularly suitable for epithelial or polarized tissues where apical–basal organization is essential (Tanaka et al., 2025). The exposure to air promotes functional specialization, such as enhanced ciliation in airway cultures or improved surface maturation in brain and gut organoids. Despite this advantage, ALI culture alters the geometry of closed-lumen organoids by forcing partial flattening or opening, which may be a limitation for studies reliant on intact lumenal architecture (Huang A. Z. et al., 2025). Sliced organoids, by necessity, compromise spherical geometry and internal compartmentalization. However, this loss of full 3D topology is counterbalanced by increased experimental accessibility (Qian et al., 2020). Slicing exposes previously inaccessible inner layers such as developing neuronal circuits, crypt-like domains, or tumor stromal niches facilitating direct visualization, manipulation, and functional recording (Chartrain et al., 2025). Structural uniformity across slices may improve the consistency of specific experimental readouts, particularly those related to controlled tissue thickness, oxygen exposure, and accessibility of internal regions (Qian et al., 2020; Petersilie et al., 2024). In addition, generating multiple slices from a single organoid enables intra-organoid replication, which can reduce variability within experimental comparisons and facilitate higher-throughput experimental designs (Chartrain et al., 2025). However, global reproducibility in organoid systems remains constrained by upstream biological variability, including donor-specific effects, differentiation heterogeneity, and protocol sensitivity (Andrews and Kriegstein, 2022; Urrestizala-Arenaza et al., 2024). Emerging ALI–microfluidic platforms further extend these structural paradigms by preserving intact three-dimensional architecture while introducing geometric confinement and controlled perfusion, enabling improved size uniformity and metabolic stability without physically opening the tissue core (Ao et al., 2020).

### 4.3 Impacts on maturation and functional development

Both ALI and slicing promote enhanced maturation by mitigating hypoxia, but they do so in different temporal and functional contexts. ALI cultures support long-term tissue development, allowing organoids to grow thicker and maintain viability for months while continuing to differentiate along physiologically relevant trajectories (Giandomenico et al., 2019). This long-term stability has proven particularly powerful in neuronal organoids, where extended timelines are necessary for synaptogenesis, circuit refinement, and glial development (Giandomenico et al., 2019). Organoid slices, due to their thin geometry, accelerate short to mid-term maturation. Cells within slices often undergo more uniform differentiation, and gradients in gene expression or metabolic activity may become reduced. This synchronization creates a more controlled system for mechanistic developmental studies and enhances the interpretability of dynamic assays such as electrophysiology, calcium imaging, or drug-response profiling (Huang J. et al., 2025). Complementing these approaches, ALI–microfluidic platforms further support functional maturation in intact organoids by integrating controlled perfusion with atmospheric oxygen exposure, enabling stable long-term electrophysiological activity and the emergence of coordinated neuronal network activity, as evidenced by spontaneous firing patterns and functional connectivity assessed through electrophysiological and multi-electrode array (MEA) recordings, dynamically regulated culture conditions.

### 4.4 Experimental accessibility and technical utility

One of the defining differences between these platforms lies in their experimental versatility. ALI cultures provide direct access to the tissue surface, enabling controlled drug application and functional imaging, making them particularly suitable for studying neuronal maturation and surface-accessible neural responses. In contrast, sliced organoids offer unparalleled access to the interior microenvironment. Techniques that are challenging in intact tissues including whole-slice patch-clamp electrophysiology, optogenetic interrogation, spatial transcriptomics, microinjection, and high-resolution live imaging become readily feasible (Landry et al., 2023). Moreover, generating multiple slices from a single organoid increases experimental throughput and enables internal replication while minimizing biological variability across samples.

ALI–microfluidic platforms further extend experimental accessibility by integrating controlled perfusion with atmospheric oxygen exposure, enabling dynamic compound delivery, real-time functional monitoring via microelectrode arrays, and improved regulation of biochemical gradients in intact neural tissues (Ao et al., 2020; Tian et al., 2024). These hybrid systems facilitate scalable neurotoxicity screening and network-level functional assessment under physiologically regulated conditions. Despite their strengths, each platform has limitations that shape optimal use cases. ALI cultures may not fully recapitulate tissue environments that depend on enclosed luminal architecture, mechanical confinement, or fluid shear stress, as exposure to air and predominantly static conditions can alter native biomechanical cues (Wörsdörfer and Ergün, 2021; Ji and Sun, 2024). Comparative studies also indicate that certain ALI configurations impose practical constraints, including extended culture durations and altered cellular behaviours relative to alternative organoid models (Zhang et al., 2025). Sliced organoids, while experimentally powerful, are less suitable for long-term studies, as thin sections can lose structural stability, undergo edge-associated degeneration, and exhibit slicing-induced stress responses (Chartrain et al., 2025). Additionally, the loss of intact three-dimensional architecture limits their capacity to model complex neurodevelopmental morphogenetic processes. In parallel, biological engineering approaches such as vascularization have emerged as a promising strategy to overcome diffusion limitations, with studies demonstrating improved maturation, reduced hypoxia, and the formation of vascular-like networks within organoids. However, these approaches remain technically complex, lack standardisation, and are not yet widely integrated into ALI-based systems, and thus represent an important direction for future development rather than a fully established solution. A comparative summary of ALI-based oxygenation strategies, highlighting their respective advantages, limitations, and experimental applicability, is provided in Table 2.

**TABLE 2:** Comparative summary of ALI-based oxygenation strategies for human brain organoids.

Category	ALI-intact organoids	ALI-sliced organoids	ALI–Microfluidic platforms	References
Oxygen Delivery	Surface oxygenation; diffusion limits persist centrally	Uniform oxygenation via reduced thickness (100–300 µm)	ALI + perfusion improves delivery; partial hypoxia persists in large tissues	Giandomenico et al. (2019), Ao et al. (2020), Qian et al. (2020), Tian et al. (2024)
Architecture	Preserved 3D geometry	Local cytoarchitecture retained; global topology disrupted	Intact 3D with geometric confinement	Ao et al. (2020), Chartrain et al. (2025)
Maturation	Supports long-term neuronal maturation	Accelerated, synchronized differentiation	Supports functional maturation and network activity	Giandomenico et al. (2019), Tian et al. (2024)
Accessibility	Surface access for imaging/drug exposure	Direct internal access (patch-clamp, imaging, omics)	MEA integration; dynamic compound delivery	Qian et al. (2020), Tian et al. (2024)
Viability	High vs. submerged	High but time-limited	Improved vs. submerged	Giandomenico et al. (2019), Ao et al. (2020)
Throughput	Moderate	High (multiple slices/organoid)	Moderate–high (parallel microfluidics)	Ao et al. (2020), Chartrain et al. (2025)
Complexity	Moderate	High (slicing required)	High (custom devices/perfusion)	Papamichail et al. (2025)
Key Limits	Central diffusion constraints	Loss of intact 3D architecture	Technical complexity; incomplete metabolic rescue	Castiglione et al. (2022)

## 5 Limitations and future directions

Brain organoid models have substantially advanced the study of human brain development, circuit formation, and neurological disease. However, each oxygenation strategy presents intrinsic biological and practical limitations that must be considered when interpreting experimental outcomes. Apical-exposure systems, including air–liquid interface (ALI) configurations, improve oxygenation and experimental accessibility at the tissue surface but remain constrained in their ability to fully recapitulate the complex internal spatial organization of the developing brain. Enclosed ventricular-like zones, deep radial architecture, and long-range cytoarchitectural patterning are not uniformly accessible in intact ALI-cultured organoids, and altered boundary conditions at the air-exposed surface may impose non-physiological stresses that influence neuronal maturation or regional identity. Critically, physiological oxygen levels in the human brain are substantially lower than atmospheric conditions (∼21% O_2_), and exposure to higher oxygen levels in standard culture systems can introduce non-physiological effects, including altered cellular responses and oxidative stress (Barakat et al., 2025). Although air–liquid interface (ALI) culture enhances oxygen diffusion and reduces hypoxic core formation, it does not reflect the native brain environment, which is not exposed to air, therefore, ALI should be considered primarily as a technical strategy rather than a physiologically accurate configuration, and its non-physiological boundary conditions should be taken into account when interpreting experimental outcomes.

Sliced cerebral organoid cultures effectively address diffusion-limited hypoxia by exposing internal tissue compartments, thereby enhancing metabolic stability and experimental access. However, these advantages are accompanied by disruption of intact three-dimensional geometry and long-range tissue continuity. Mechanical perturbation introduced during slicing can elicit cellular stress responses, and thin organoid sections typically exhibit reduced longevity compared with intact constructs. Although vibratome-based slicing improves oxygen diffusion, it is typically performed at low frequency (e.g., approximately once per month) to minimise cumulative mechanical stress, as it may cause localized disruption to neuronal processes and induce transient injury-related responses that should be considered when interpreting experimental outcomes. In addition, loss of native tissue geometry may impair the establishment of physiological biomechanical and signalling gradients that are critical for morphogenetic processes and circuit-level organization. ALI–microfluidic platforms partially bridge these limitations by integrating atmospheric oxygen exposure with controlled perfusion and geometric confinement, enabling improved size uniformity, metabolic support, and functional readouts in intact organoids. Nevertheless, these systems remain technically complex, lack standardisation across laboratories, and do not yet fully resolve diffusion constraints in larger tissues.

Beyond biological fidelity, scalability remains a critical consideration for translational and high-throughput application brain organoid platforms. Widely used industry-standard platforms such as orbital shakers and spinning bioreactors enable scalable organoid production through enhanced convective mixing and parallel culture formats. However, despite their suitability for high-throughput applications, these systems remain constrained by intrinsic oxygen diffusion limitations within larger three-dimensional tissues. Intact ALI cultures, while supporting prolonged maturation, are inherently space-intensive and limited by one-organoid-per-insert formats, restricting parallelization. Sliced organoid approaches improve experimental throughput by enabling multiple sections to be generated from a single organoid, facilitating internal replication and multiplexed assays, although at the expense of long-term structural integrity. In contrast, emerging ALI–microfluidic systems offer greater potential for scalable organoid production through array-based confinement and perfusion, enabling size-uniform tissue generation, parallel functional readouts, and compatibility with automated compound screening. However, widespread adoption remains constrained by device complexity, fabrication requirements, and a lack of standardised workflows. Addressing these engineering barriers will be essential for translating enhanced oxygenation strategies into robust, high-throughput platforms suitable for disease modelling and therapeutic discovery. Miniaturization of organoids represents an alternative strategy to improve oxygen and nutrient diffusion by reducing tissue size and diffusion distance. However, while smaller organoids may exhibit improved metabolic uniformity and reduced hypoxia, this approach may limit the development of higher-order tissue architecture, long-range connectivity, and advanced maturation. Therefore, miniaturization alone is unlikely to fully recapitulate the structural and functional complexity of the human brain, highlighting the need for complementary strategies such as ALI, slicing, and perfusion-based systems.

In addition to oxygen and metabolic constraints, the maturation of brain organoids is also influenced by the biochemical and cellular composition of the culture system. Current protocols may lack essential growth factors, extracellular matrix components, and supporting cell types that contribute to tissue development *in vivo*. Incorporating these missing signals, including cell–cell and cell–matrix interactions, represents an important complementary strategy to enhance organoid maturation beyond improvements in oxygen delivery alone.

## 6 Conclusion

Engineering approaches that improve oxygen delivery, including air–liquid interface (ALI) culture, organoid slicing, and emerging ALI microfluidic platforms, have significantly strengthened cerebral organoid models by reducing hypoxia, extending culture longevity, and supporting higher-order neuronal maturation and functional analysis. ALI enhances surface oxygenation while preserving tissue architecture, slicing directly relieves diffusion barriers by exposing internal regions, and ALI microfluidic systems add controlled perfusion to better regulate nutrient and oxygen transport in intact constructs. Despite these advances, each strategy involves trade-offs between oxygen delivery, three-dimensional organization, experimental accessibility, and scalability, highlighting the need for hybrid platforms that combine ALI, controlled sectioning, perfusion, and microphysiological engineering.
